# Understanding consumer behaviour in small waste electrical and electronic equipment collection: Insights from Australia

**DOI:** 10.1177/0734242X251334242

**Published:** 2025-04-25

**Authors:** Gimhan Jayasiri, Sunil Herat, Prasad Kaparaju

**Affiliations:** School of Engineering and Built Environment, Griffith University, Nathan, QLD, Australia

**Keywords:** Small WEEE, collection, WEEE, consumer behaviour, management

## Abstract

Production growth of electrical and electronic equipment (EEE) has led to a significant increase in waste electrical and electronic equipment (WEEE), with small EEE having the highest generation rate but the lowest formal collection rate globally. In Australia, many consumers tend to stockpile old small EEE, which limits collection rates and reduces the potential for resource recovery. This study aimed to assess consumer behaviour related to the collection of small EEE in Australia through a multivocal literature review and an online survey of 403 respondents. The analysis revealed that consumers are disposing of small WEEE along with general household waste, and most are uncertain of the correct disposal method. Almost half of the respondents are willing to pay to manage small WEEE and prefer to drop them at a designated location. Hence, to increase the collection rates, this study recommends setting realistic collection targets based on products on the market rather than waste generation estimates based on average lifespan. In addition, correct disposal can be encouraged by integrating small WEEE in kerbside collection and providing incentives. In order to strengthen this, awareness campaigns should target all age and income groups to increase collection rates and product circularity.

## Introduction

The global digital transformation has increased the production of electrical and electronic equipment (EEE), leading to a rise in waste electrical and electronic equipment (WEEE) containing both valuable and hazardous materials. WEEE has increased by 2300 kilotonnes (kt) annually, reaching 6.2 megatonnes globally in 2022. However, only 22% was formally collected and recycled (Baldé et al., 2024), with the rest managed by the informal sector (Afroz et al., 2013; Islam et al., 2016) or discarded with municipal waste (Pérez-Belis et al., 2017; Saritha et al., 2015). This indicates that most of the valuable materials in WEEE are not recycled, whereas hazardous materials may have caused harm to the ecosystem due to incorrect disposal (Liu et al., 2023). Among the various categories of WEEE, small EEE, which mainly includes household appliances and tools, has the highest generation rate but a low formal collection rate of only 12% by weight (Baldé et al., 2024). When the collection is not correctly aligned with the generation rates, consumers stockpile the small WEEE in their households (Casey et al., 2019). Since the dimensions of the small WEEE do not exceed 20 cm, consumers have found it convenient to dispose of along with regular household waste. Collection efforts for small WEEE are also less appealing, as most collecting organisations try to omit them due to the low financial benefits (Habib et al., 2022).

Following a pattern similar to the global trends, most small EEE in Australia are low-value items with a shorter life expectancy. In 2019, small WEEE accounted for one-quarter of the total WEEE generated in Australia. Still, this figure is estimated to nearly double by 2035 (Department of Agriculture and Water and Environment, 2021). In comparison to the European Union, which has reasonable WEEE collection rates (Islam et al., 2018; Rotter et al., 2011), Australia has a limited number of takeback schemes, and even the existing ones are not focused on collecting small EEE (Morris and Metternicht, 2016). Hence, most consumers stockpile small EEEs or dispose of them incorrectly. This has led to low collection rates (Golev and Corder, 2017), reducing the potential for resource recovery (Golev et al., 2016). Consumers not being legally obliged to drop off small EEE responsibly has also fuelled the low collection rates in Australia (Islam and Huda, 2020). In addition, regulatory bodies are also facing barriers like budget restrictions (Morris and Metternicht, 2016) and inadequate storage (Dias et al., 2018), which has further hindered small WEEE collection. Given this background, the following research gaps related to the small WEEE collection in Australia were identified.

RG1: What are the average lifespans of small EEE in Australia?
RG2: What is the status of the small EEE when being replaced?
RG3: Are consumers willing to pay for small WEEE management?
RG4: What are the preferred methods of small WEEE disposal?

To answer these questions, a consumer questionnaire survey was then administered to gather consumer experience and opinions. Based on the literature review and the consumer survey analysis, recommendations were made to improve small WEEE collection in Australia.

## Literature review

The existing literature related to the small WEEE collection was searched using the advanced search tool in the Scopus database. The search query used was ( ( TITLE-ABS-KEY (‘small EEE’) OR TITLE-ABS-KEY (‘small household appliances’) OR TITLE-ABS-KEY (‘small WEEE’) OR TITLE-ABS-KEY (‘small Equipment’) ) AND TITLE-ABS-KEY (collection ) ) which revealed 29 articles. Then, the review was expanded further to a multivocal literature review (Garousi et al., 2019) to include first and second-tier grey literature, which included technical reports, guidelines and working papers. This was done using the Google advanced search tool, and based on the page ranking, highly relevant documents were selected for initial analysis. After studying the abstracts and executive summaries, 45 documents were selected for detailed analysis.

### Definition of small EEE

Various WEEE classifications exist (Duan et al., 2016; Kahhat et al., 2008; Kumar et al., 2017), with the most widely adopted being Europe’s WEEE Directive (Shittu et al., 2021). Out of the six categories covered in the EU WEEE Directive, small EEE or ‘small equipment’ are classified as products with ‘no external dimension more than 50 cm’. Building on this, the United Nations University (UNU) aligned Harmonised Statistical codes and identified 20 subcategories of appliances as small EEE (Forti et al., 2018). However, in the Australian context, the scope of small EEE was identified as the ‘products found in homes and small businesses, weighing up to 20 kg’, which also included IT and Telecommunication products (Department of Climate Change, Energy and the Environment and Water, 2023). After comparing the subcategories in both the EU and Australian contexts, the authors selected 17 categories indicated in Table 1 as small EEEs for this study, excluding small IT, telecom and mobile phones.

**Table 1.** Small EEE considered in this study.

Reference name	List of common appliances	Average life span (years) Forti et al. (2020)
(0114) _Microwaves	Microwave ovens	15.9
(0201) _Clocks, Watches, Irons, fans	Small ventilators, irons, clocks	7.5
(0202) _Food Preparation EEE	Toasters, grills, frying pans	9.8
(0203) _Hotwater EEE	Coffee and tea makers	7.2
(0204) _Vacuum Cleaners	Vacuum cleaners (professional ones are excluded	9.9
(0205) _Personal Care	Electric toothbrushes, hair dryers and razors	7.6
(0401) _Consumer EEE	Headphones and remote controls	9.1
(0402) _Portable AV	MP3 players, car-navigation equipment	9.0
(0403) _Music, Radio Instrument	Electric music instruments, radio and audio sets	8.9
(0404) _Video Players	Blue Ray players, DVD players	7.9
(0405) _Speakers	Speakers (single and mounted)	12.0
(0406) _Cameras	Digital cameras and Video recorders	6.4
(0601) _Household Tools	Electric drills, saws, lawn mowers	13.3
(0701) _Toys	Different kinds of electric toys, including drones	4.1
(0702) _Game Consoles	Home, handheld and hybrid video game consoles	4.6
(0801) _Household Medical EEE	Instruments to monitor blood sugar and blood pressure	11.9
(0901) _Household Monitoring EEE	Different kinds of sensors and alarms (smoke, heat)	5.3

EEE: electrical and electronic equipment.

### Small WEEE generation and collection: Global scenario

When an owner or user decides an electronic device is no longer needed, it becomes WEEE. This usually happens when the device can no longer serve its functional purpose. At this point, the owner may attempt to repair it, but if repair is not viable, the non-functional device is disposed of, entering the waste stream where it is either recycled or sent to a landfill. Alternatively, a user may lose interest in a still-functional device due to discontinued software updates or the release of newer technology (Islam et al., 2020). In such cases, the device may be stockpiled, reused or shared with another party, but it will still end up in the waste stream.

A study in Ireland highlighted some common behaviours regarding small WEEE. Many consumers store unused devices in hidden places, such as behind picture frames or drawers, often due to difficulty categorising them. Some keep these items for potential future use, whereas those with technical skills tend to repair and extend their lifespan. However, peripherals are frequently discarded as general waste, and dropping off small WEEE at retailers is a rare scenario. As a result, small WEEE often goes unnoticed in homes, with over 80% of Irish households storing unused EEE (Casey et al., 2019). Stockpiling is also common in other regions (Fraige et al., 2012; Miliute-Plepiene, 2021; Ylä-Mella et al., 2014), and low formal collection rates for small EEE fuel this scenario. Hence, in countries like Malaysia, people tend to sell the small WEEE to informal collectors as they are aware of the precious materials in small WEEE (Afroz et al., 2013; Islam et al., 2016), whereas in most countries, they often get disposed of along with general waste (Dagiliūtė et al., 2019; Habib et al., 2022; Nowakowski, 2016; Pérez-Belis et al., 2017; Saritha et al., 2015; Trihadiningrum et al., 2023).

To increase the collection rates, countries adopt different mechanisms. These strategies vary based on economic capacity, and the most environmentally friendly strategy is not always cost-effective (Hanafi et al., 2008). Currently, a variety of collection strategies are followed globally. The Drop-off method requires consumers to deliver small WEEE to collection boxes placed in high-traffic areas or to return them to the retail stores where the items were initially purchased (Hanafi et al., 2008; Kang et al., 2020; Melissen, 2006; Ministry of Environment, 2018; Nowakowski et al., 2021; Rotter et al., 2011). This method is effective when collection points are located in areas with high population density (Habib et al., 2022). The involvement of retailers has also increased the collection rates in this method (Magalini and Huisman, 2006). Bringing-in method is another mechanism that requires upgrading municipal collection facilities to handle small WEEE. Additional staff can be employed to ensure proper collection. This approach also reduces the risk of theft during disposal (Ministry of Environment, 2018). However, the door-to-door method is the most convenient for consumers, as municipal councils may organise periodic or on-call kerbside collection events (Casey et al., 2019; Hanafi et al., 2008; Ministry of Environment, 2018; Rotter et al., 2011; Solé et al., 2012). Even though it may be costly for the councils, it can be offset by adding a small fee to the price (Islam et al., 2018). In the Pick-up method practised in Japan, small WEEE is disposed of as general waste but recovered from the conveyor belt before processing at a Material Recovery Facility or recycling plant (Ministry of Environment, 2018). In addition to the above methods, the Return-flight method is another collection technique that uses product delivery services, where retailers collect small WEEE during deliveries and return them to the store. This approach is also helpful for online sales (Ministry of Environment, 2018; Rotter et al., 2011), making the sellers accountable for the safe disposal of their products. All these methods should be assessed appropriately during implementation as the convenience and cost largely depend on the geographical location.

When these mechanisms exist, given the nature of small WEEE, consumer incentives are suggested further to enhance participation (Duygan and Meylan, 2015). A recent study done in Europe has identified three types of incentives. Reward-based incentives offer users several forms of compensation and proper disposal. Convenience-based incentives focus on timesaving via services like pick-up options and drop-off points. Other incentives are often tied to charitable causes, contests or raising awareness about end-of-life costs, which may include visible fees to the consumer, promoting greater responsibility and engagement in the recycling process (Rebecca Bliklen and Felicitas Frick, 2023).

The government, producers or recycling enterprises can lead collection schemes for small EEE. In the Producer-Led scheme, original equipment manufacturers or their retailers manage collection under government supervision, whereas recyclers are responsible for recovering materials. The Government-Led scheme empowers local governments to organise the collection, with producers focusing on using recovered materials. In the Recycler-Led scheme, recyclers handle both the WEEE collection and profits from recovered materials. Due to the size of the small EEE, specialised treatment technologies are needed, and improving access thresholds can boost collection efficiency (Liu et al., 2018). However, the current global trend is for producer-led schemes to ensure extended producer responsibility (EPR). Switzerland is one of the nations that follow EPR and has a higher WEEE collection rate. Importers and manufacturers cover the physical and financial aspects of the system, whereas consumers are obliged to return the end-of-life products irrespective of the brands and product types. In the Swiss System, multi-level monitoring is also implemented to avoid free riders by closely monitoring the downstream to ensure environmental standards (Islam et al., 2018).

In addition to a suitable collection scheme, transportation distances and the size of the collection points also affect the collection rates. One of the main challenges faced by Finland in establishing a nationwide collection network is the long transportation distances and sizes of the collection points. Physical spaces of the collection slot/cages are limited, and the WEEE returns have been increased due to the receipts of sudden larger batches from rural households. Hence, a decentralised collection network with pretreatment and sorting facilities, even in the slightest points, would be ideal since the advantage of recycling depends mainly on the distance travelled to collect the WEEE (Queiruga et al., 2012). A study done in Japan has identified that to achieve a maximum collection rate, the distance between collection points should be 1 km maximum (Mishima and Nishimura, 2016).

Furthermore, when unofficial collection points receive valuable WEEEs, official parties lose the system’s cost-effectiveness. Mobile collection systems are also a suitable option for supporting the collection process in the least populated regions (Ylä-Mella et al., 2014). Due to the complexities arising from the different stakeholder engagements in collection schemes, clearing houses have been established to streamline communication between collectors and consumers (Rotter et al., 2011).

### Small WEEE generation and collection: Australian scenario

According to a recent study on small WEEE in Australia, small WEEE is expected to reach 368 kt per year in 2050 from 197 kt per year in 2025. Out of the appliances mentioned in Table 1, waste generation rates of clocks, watches, irons, portable audio and video players and Blu-Ray and DVD players are declining, which is mainly due to the replacement of these products by appliances in other categories due to the technological advancements (Jayasiri et al., 2024). Most of the small EEE are disposed of due to the lower lifespans and limited firmware updates (Productivity Commission, 2021). Furthermore, current regulated recycling schemes in Australia do not collect small WEEE (Morris and Metternicht, 2016), which has forced the consumers to hoard (Golev et al., 2016) or dispose of the small EEE alongside the Kerbside collection, which ends up in landfills (Golev et al., 2019). The country has minimal takeback schemes as some councils have advised the customers to drop off their small EEE at nearby retail stores and resource recovery centres (Brisbane City Council, 2023). Providing reasonable access to collection programmes is recommended in previous studies, especially in regional areas (Davis and Herat, 2008; Morris and Metternicht, 2016). Consumers are also not legally obliged to drop off small EEE, making collecting much harder (Islam and Huda, 2020). Budget restrictions (Morris and Metternicht, 2016) of the councils and improper storage (Dias et al., 2018) are some other barriers Australian governing bodies face in collecting small WEEE.

## Materials and methods

**Figure 1.**
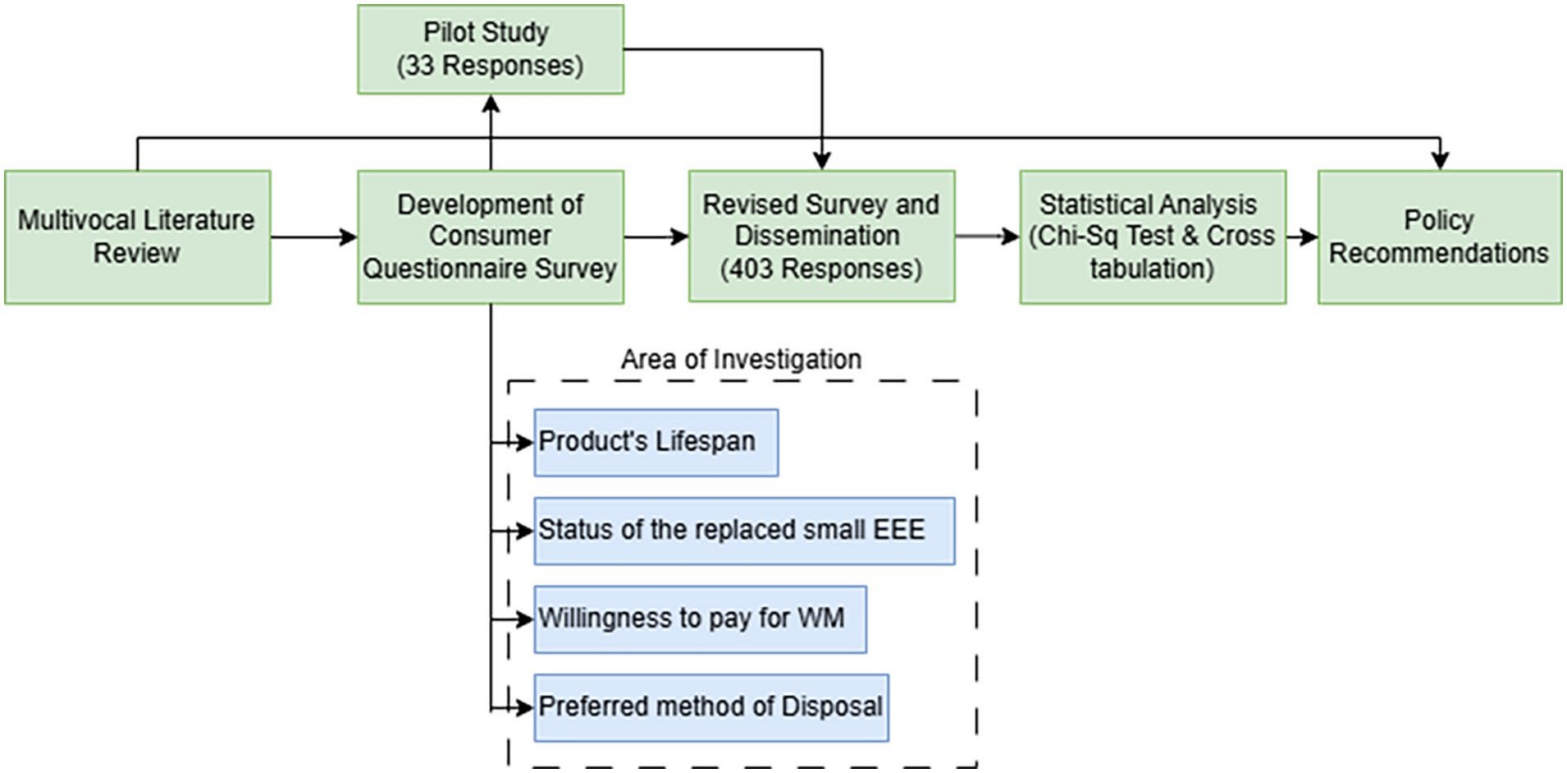
Research process followed in this study.

After the literature review, a questionnaire survey was employed to identify consumer behaviour related to small EEE. Following the research gaps identified, this study focused on four aspects: the product’s lifespan, the status of the replaced small EEE, consumer willingness to pay for waste management and preferred disposal methods. The questionnaire was developed concerning previously published WEEE-related survey studies and with the insights from the literature review (Bovea et al., 2018; Duke et al., 2018; Genever et al., 2018; Habib et al., 2022; Islam et al., 2021; Pérez-Belis et al., 2017; Shittu et al., 2022). The survey questions and related references are detailed in Annex 1. Data collection commenced on 11 December 2023, and concluded on 12 August 2024, covering 8 months. The survey was designed and distributed electronically via the Qualtrics platform. Data reliability were ensured by incorporating bot detection and ambiguous text filters. The GeoIP tracking feature was also used to track the IP addresses of the respondents to ensure that the responses were limited only to Australia. The collected responses were then analysed statistically using the R programming language (GNU General Public License). The research process followed in this study is indicated in Figure 1.

### Survey design

Given the wide range of products in the small EEE category, 21 commonly used products were selected to get the consumer experience. The inclusion criteria for the survey were consumers who have replaced any of these products identified in Australia. An academic expert in the field validated the initial version of the questionnaire survey. Then, a pilot study with 33 participants was conducted to understand whether the consumer survey questions were appropriate and less confusing. This was done based on the recommendations of Krosnick (2018). In addition, the ‘ExpertReview’ functionality was also used to ensure that the survey is user-friendly. Then, based on the feedback from the pilot study, the final version of the consumer survey was prepared and disseminated online.

### Selection of the sample

Since the survey is targeted at the users of small EEE in Australia, the sample size for the survey was calculated based on the following equation (1) (Kotrlik and Higgins, 2001). This equation was also used in previous WEEE-related survey studies conducted for larger populations (Bovea et al., 2018; Islam et al., 2021; Pérez-Belis et al., 2017)

(w1) $$n=\frac{z^{2}p\left(1-p\right)}{d^{2}}$$

where n is the sample size, and for a 95% confidence interval, (z) was selected as 1.96. The *p*-value is the expected prevalence, which was assumed to be 0.5, and the margin of error (d) was 0.05. Since the study is conducted in all states of Australia, it was noted that a sample of 385 was appropriate for the survey. This was similar to a previous study done for WEEE in Australia (Islam et al., 2021). Hence, for the survey, the selected sample size was 400.

### Distribution of the survey

The final version of the questionnaire survey was distributed across various online platforms to capture a broad range of respondents. The survey was posted mainly on Facebook community groups representing different suburbs across all states of Australia. It was also posted on the Griffith University website and featured in newsletters of various professional organisations. All the respondents were over 18, and their participation was voluntary. A total of 403 responses were collected, and two incomplete responses were removed from the final analysis.

### Statistical analysis

The association between the independent variables, such as age, state, education level and total annual household income and the dependent variables, such as the status of the old product and willingness to pay for waste management and disposal methods, were checked during the statistical analysis. A chi-square test of independence was done to determine the significance levels of the relationship, which was also used in previous WEEE-related studies (Islam et al., 2021; Pérez-Belis et al., 2015). In addition, cross-tabulation analysis was also used to identify the association of different categorical variables with socio-demographic variables. All statistical processing was performed using the R programming language.

## Survey analysis

### Demographic characteristics

**Figure 2.**
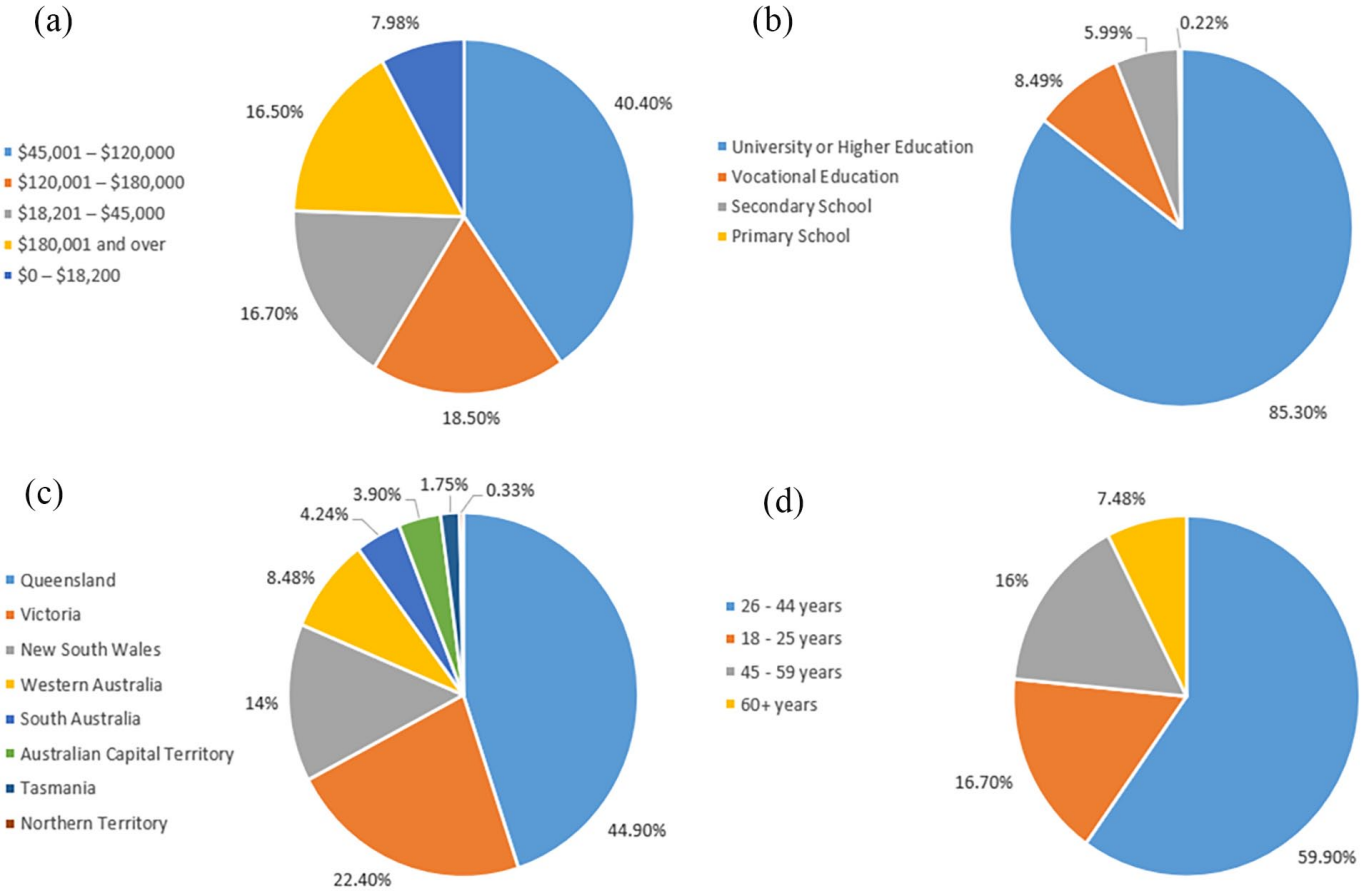
Annual average household income (a), Education level (b), state (c) and the age (d) of the respondents.

The survey received a total of 401 valid responses, and the majority of the responses (59.9%) were from the age group 26–44 years. 85.3% of the respondents have attended a university or higher education institute. When considering the geographical location of the respondents, 44.9% were from Queensland, followed by Victoria (22.4%) and New South Wales (14%). When looking at the Average annual household income of the respondents, 18.5% belonged to the income category of $120,001 to $ 180,000, followed by $18,201 to $45,000 and $180,001 and over, with approximately equal representation around 16.5%. These socio-economic conditions are shown in Figure 2.

### Descriptive analysis

#### Lifespan of small EEE

**Figure 3.**
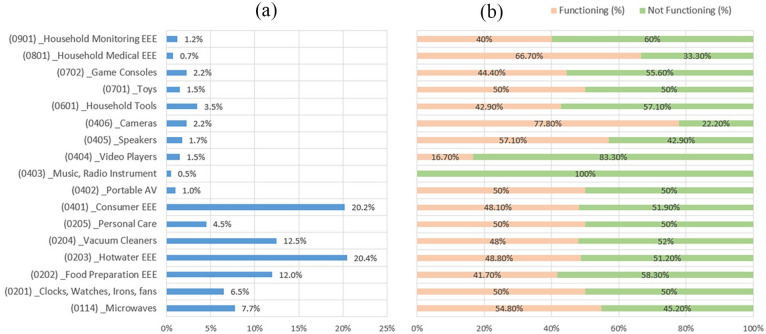
Small EEE replaced (a) and their status during the replacement (b). EEE: electrical and electronic equipment.

Small WEEE collection rates are directly associated with the small WEEE generation rates. The lifespan of small EEE is an essential variable in determining these rates. According to the literature, a product’s lifespan is defined as the time it remains in use, including repair and reuse, starting from its purchase date and ending when it is disposed of (Forti et al., 2018). For small EEE, consumption patterns and rapid technological advancements have led to significant variations in their lifespan, with many being disposed of before their functional life ends (Jayasiri et al., 2024). It was confirmed from the consumer survey that more than 50% of the small EEEs that were replaced were still functioning. Furthermore, none of the music and radio instruments functioned during the replacement, and cameras had the highest functioning rate among the small EEE, as shown in Figure 3. In addition, the chi-square test did not show any significant relationships between the demographic characteristics and the functionality of the replaced small EEE.

**Figure 4.**
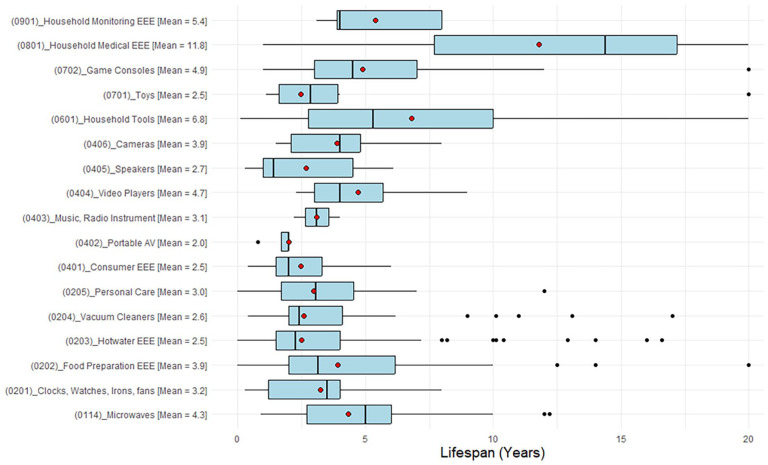
Estimated lifespan for small EEE from the consumer survey. EEE: electrical and electronic equipment.

There are different ways to incorporate the product lifespan into WEEE generation rates, and currently, the widely used method is the use of Average Lifespan, where its dynamic nature is modelled by the Weibull distribution function (Forti et al, 2018; Islam and Huda, 2019; Jayasiri et al., 2024). However, according to the best of the authors’ knowledge, currently, there are no any consumer surveys done for small EEE to get grassroots-level data on lifespans. All the WEEE generation models have used the data published for UNU for non-European countries. Hence, a new lifespan dataset is estimated from the consumer survey performed in this study, as shown in Figure 4 below.

During the analysis, it was observed that the average lifespans of most small EEE differed significantly from the UNU data, except for items such as game consoles (0702), household medical EEE (0801) and household monitoring EEE (0901). As a result, the authors chose to model the generation rates of small WEEE to assess the impact of this variation using the methodology performed by Jayasiri et al. (2024). It was noted that small WEEE generation rates are projected to increase by an additional 8–10% from previously modelled estimates, reaching approximately 234 kt per year by 2030 and up to 402 kt per year by 2050.

#### Status of replaced small EEE

**Figure 5.**
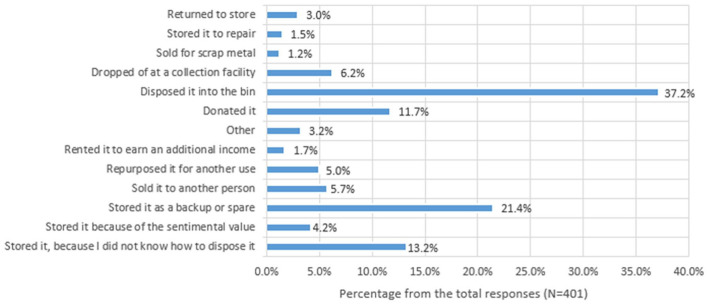
Status of old small EEE after the replacement. EEE: electrical and electronic equipment.

Figure 5 indicates that most consumers (37.2%) disposed of their old small EEE by placing it in the bin, confirming the observations from the literature. However, 21.4% stored the appliances as backups or spares, whereas 13.2% kept them due to uncertainty about proper disposal methods. Some consumers (11.7%) donated the items, which indicates their interest in product circularity. Other methods included selling to another person (5.7%), repurposing (5%) and dropping them off at collection facilities (6.2%). A smaller group rented the items for additional income (1.7%), sold them for scrap metal (1.2%) or stored them for future repair (1.5%), with only 3% returning them to the store. Some consumers also disposed of small WEEE at council WEEE collection days or WEEE collection bins at their workplaces. These findings suggest that many consumers (62.8%) are willing to divert small EEE from landfills if proper collection methods are provided.

The status of small EEE and several demographic variables, including state of residency (*p* = 0.03), education level (*p* = 0.01) and annual household income (*p* = 0.03), were found to be significantly correlated by a chi-square test. Most respondents from Queensland had either disposed of small EEE with municipal waste or were unsure about the correct disposal techniques. Similar situations exist in other states, emphasising the necessity of an extensive national collection system. In addition, some consumers in Victoria and Queensland have sold or reused their small EEEs, indicating that some are proactively attempting to divert small WEEE from landfills. Even among the educated population, it appears that many respondents with a university degree or higher still disposed of small EEE with their ordinary household waste, indicating a lack of awareness. This pattern was consistent across all income levels. However, households with the highest annual average income have followed more circular practices such as selling, donating and repurposing small EEE.

#### Willingness to pay for management of small WEEE

From the responses received, around 47% of the consumers were willing to pay to manage small WEEE. Appliances like household monitoring EEE (60%), medical EEE (66.7%), game consoles (77.8%) and toys (83.3%) had a higher proportion of consumers unwilling to pay. On the other hand, a notable percentage of respondents indicated willingness to pay for appliances like cameras (77.8%), speakers (57.1%) and vacuum cleaners (68%). Consumers are willing to pay an average of $15 to manage small WEEE. Furthermore, no significant association was observed between the demographic characteristics and the willingness to pay for waste management of small EEE.

#### Expected method of disposal

**Figure 6.**
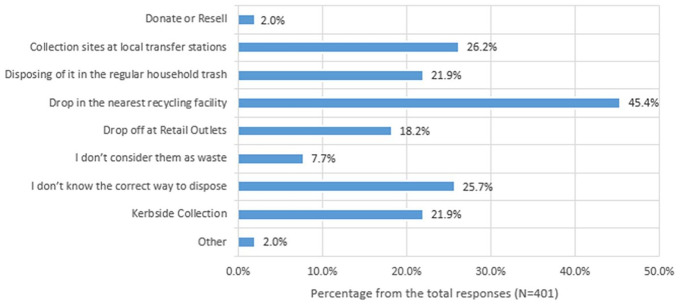
Expected method of disposing of small WEEE. WEEE: waste electrical and electronic equipment.

According to Figure 6, it is evident that most consumers (45.4%) prefer to dispose of their small EEE by dropping them off at the nearest recycling facility. Local transfer stations are the second most popular option (26.2%) for disposal, with drop-offs at retail outlets also being common (18.2%). This shows that many consumers are willing to take their small WEEE to designated locations. Furthermore, 21.9% still prefer more convenient options like kerbside collection or disposing of them in the council bin. However, 25.7% of consumers are unsure about the correct way to dispose of small WEEE. Cross-tabulation of the status of replaced products and expected disposal methods revealed that around one-third of respondents who were unwilling to use drop-off options had disposed of their old products through regular household waste. Furthermore, 7.7% of respondents do not consider small EEE as waste, whereas a small minority (2%) opt to donate, resell or use other methods for disposal.

When comparing the demographic characteristics with the expected method of disposal, a strong association was evident with the age of the respondents (*p* = 0.003). Disposing of the small EEE along with the regular household waste was common in all the age groups, even though most of them were willing to drop it off at a convenient location.

## Discussion, recommendations and policy implications

This is the first systematic study on consumer practices in using and disposing of small WEEE in Australia. Analysis of the literature review and the consumer survey reveals a strong need for an effective collection mechanism for small WEEE in the country. As the generation rates rise, limited collection mechanisms will encourage non-environmental friendly disposal behaviour like disposing of small WEEE along with regular household waste, which is evident in this study. Additionally, a significant portion of small EEEs that have been replaced are still functioning and end up in landfills. The study also reveals that many consumers are uncertain about proper disposal methods. Based on these results, the following recommendations are made to improve the small WEEE collection in Australia.

### Realistic collection targets

The generation and collection of small WEEE are two interdependent factors. Establishing a realistic collection target is essential for both Producer Responsibility Organisations and regulatory bodies. According to a recent discussion article published by the Department of Climate Change, Energy, the Environment and Water, a collection target is proposed based on small WEEE generation rates (Department of Climate Change, Energy and the Environment and Water, 2023). However, consumer data indicate that forecasting small WEEE generation using lifespan profiles from the UNU for non-EU countries may underestimate the actual levels in Australia. Therefore, it is recommended that collection targets be calculated by multiplying the number of products on the market by a particular factor. This will omit the inaccuracies of small WEEE generation estimations and will put more weight on the producers or importers. This method is also recommended in the EU WEEE Directive (Baldé et al., 2020).

### Convenient collection mechanisms

The survey shows that many consumers prefer to drop off their old appliances at specific locations like collection centres, retail stores or recovery facilities. However, most places do not accept small WEEE because a dedicated stewardship programme is not in place. Furthermore, retail stores usually do not take back small WEEE unless the appliance is exchanged for a newer product. However, the most convenient option for consumers is to dispose of their old appliances via regular household bins or kerbside collection. Since many consumers have stored old small EEE, local government councils can collect these items along with their bulk kerbside collections.

Additionally, the return-in-flight method is another approach that can be applied in regional areas and to put more responsibility on online sellers. In any case, the costs can be shared among consumers, as there is a willingness to pay for waste management. On the other hand, consumers can be incentivised for proper disposal.

### Consumer awareness campaigns

The survey indicated a significant lack of awareness among consumers regarding proper disposal methods for EEE. Educational campaigns should target all age and income groups, and this outreach can be effectively integrated during the product purchasing process. Since almost one-third of the respondents did not know the correct way to dispose of their old devices, and some even did not consider their old small EEE as waste, awareness initiatives should emphasise the environmental impacts of improper disposal. Consumers must be aware of the proper locations and disposal techniques for small EEEs, as some states have banned their disposal in landfills. Appliances can be sold for scrap metal, but this is not a long-term solution because it ignores the financial benefits of properly recovering precious metals through recycling. The results from the study indicate that consumers are interested in practising circular economy principles, where many have repurposed, resold or stored old devices as spares. However, eventually, these items will reach the end of their usable life and require disposal. Therefore, awareness campaigns should target increasing collection rates as well as product circularity.

## Conclusions

This study highlights critical gaps in the collection of small WEEE in Australia. Increasing generation rates and inadequate collection systems fuel non-environmental friendly disposal behaviours such as discarding small WEEE in regular household waste. Many replaced small EEE that are still functional end up in landfills due to the consumer’s uncertainty about proper disposal methods

To address these issues, the study recommends establishing realistic collection targets based on products on the market rather than lifespan-based estimates, implementing convenient collection mechanisms like kerbside pick-up or drop-off points and enhancing consumer awareness through targeted educational campaigns. These measures can improve collection rates and promote a circular economy by maximising resource recovery. These actions can help Australia build a more sustainable and efficient system for collecting small WEEE by fostering collaboration among stakeholders, including consumers, producers and policymakers.

### Limitations and future research

The primary aim of this study was to identify broad consumer practices and trends in using and disposing of small WEEE in Australia. Techniques like chi-square tests and cross-tabulation analysis were chosen because of their simplicity, as they align well with the exploratory nature of the research, providing straightforward associations between key variables. Future studies could incorporate more advanced statistical techniques like logistic regression analysis, multivariate analysis and structural equation modelling to examine complex relationships between the variables for richer datasets. In addition, interviews and focus group discussions are also recommended as future work to understand motivations and attitudes influencing disposal behaviours.

## Supplemental Material

sj-docx-1-wmr-10.1177_0734242X251334242 – Supplemental material for Understanding consumer behaviour in small waste electrical and electronic equipment collection: Insights from Australia Supplemental material, sj-docx-1-wmr-10.1177_0734242X251334242 for Understanding consumer behaviour in small waste electrical and electronic equipment collection: Insights from Australia by Gimhan Jayasiri, Sunil Herat and Prasad Kaparaju in Waste Management & Research
