# Supplementary material for: Understanding consumer behaviour in small waste electrical and electronic equipment collection: Insights from Australia
Source: Waste Manag Res. 2025 Apr 25;43(10):1548–58. doi: 10.1177/0734242X251334242 (PMC12476481; doi:10.1177/0734242X251334242)
Supplement: sj-docx-1-wmr-10.1177_0734242X251334242 – Supplemental material for Understanding consumer behaviour in small waste electrical and electronic equipment collection: Insights from Australia [file sj-docx-1-wmr-10.1177_0734242X251334242.docx]

Q1 is displayed as the information sheet

Q2 Select any product from the following list that you have replaced during your stay in Australia.

- Microwave Oven
- Table/ Floor Fan
- Battery Operated Clocks
- Food Grinder/ Mixer
- Electric Toaster
- Coffee Machine
- Air Fryer
- Rice Cooker
- Vacuum Cleaner
- Hair Dryer
- Headphones
- Portable Audio Players
- Radios
- DVD/ Blue Ray Player
- Speakers
- Camera
- Any small Power Tool (Drill, Saw, Lawn mower)
- Any small Electric Toy
- Video Game Console
- Hearing Aid
- Fire Alarm
- I haven't replaced any of these products

**End of Block: Elegibility**

**Start of Block: Repair and Reuse**

Q3 Was it still functioning during the time of replacement? (Duke et al., 2018; Genever et al., 2018)

- Yes
- No

Page Break

*Display This Question:*

*If Q3 = Yes*


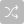


Q4 What was the reason for the replacement? (Select all that apply) (Duke et al., 2018; Genever et al., 2018)

- It was out of fashion.
- New product had more features
- It was too old
- Other:

Page Break

*Display This Question:*

*If Q3 = No*


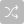


Q5 What was the main reason for the replacement? (Select all that apply) (Duke et al., 2018; Genever et al., 2018)

- I tried to repair it by myself, but it didn’t work
- I tried to repair it from a technician/ repair shop, but it didn't work
- New product was cheaper than the repair costs for old product
- It was still under warranty, so I received a replacement.
- I did not consider repair
- Other:

Page Break

Q8 For how long **(in years)** you used these products before replacement?(Islam et al., 2021; Bovea et al., 2018)

Drag the bar to indicate the number of years

| 0 | 4 | 8 | 12 | 16 | 20 |
| --- | --- | --- | --- | --- | --- |

| Microwave Oven | 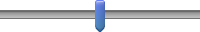 |
| --- | --- |
| Table/ Floor Fan | 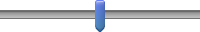 |
| Battery Operated Clocks | 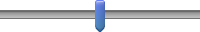 |
| Food Grinder/ Mixer | 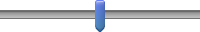 |
| Electric Toaster | 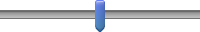 |
| Coffee Machine | 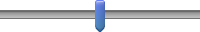 |
| Air Fryer | 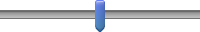 |
| Rice Cooker | 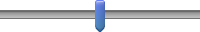 |
| Vacuum Cleaner | 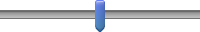 |
| Hair Dryer | 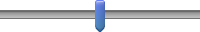 |
| Headphones | 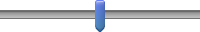 |
| Portable Audio Players | 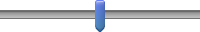 |
| Radios | 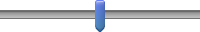 |
| DVD/ Blue Ray Player | 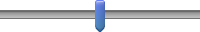 |
| Speakers | 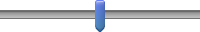 |
| Camera | 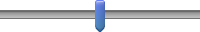 |
| Any small Power Tool (Drill, Saw, Lawn  mower) | 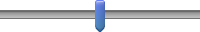 |
| Any small Electric Toy | 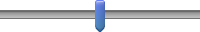 |
| Video Game Console | 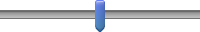 |
| Hearing Aid | 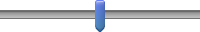 |
| Fire Alarm | 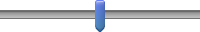 |


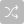


Q9 What did you do with your old product? (Select all that apply) (Habib et al., 2022; Genever et al., 2018; Duke et al., 2018; Islam et al., 2021; Shittu et al., 2022; Pérez-Belis et al., 2017; Bovea et al., 2018)

- Repurposed it for another use.
- Stored it as a backup or spare
- Stored it because of the sentimental value
- Stored it, because I did not know how to dispose it
- Sold it to another person.
- Rented it to earn an additional income.
- Donated it
- Disposed it into the bin
- Other:

Page Break

**Start of Block: Waste Disposal**

Q18 How much extra are you willing to pay for the waste management of the above product? (AUD per Product) (Duke et al., 2018)

Drag the bar to indicate the amount

0 20 40 60 80 100

AUD
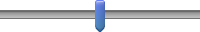


Page Break

Q21

Please mention any other experience you had with above products

**End of Block: Waste Disposal**

**Start of Block: Demographic Data**

Q22 What is your Age Group?

- 18 - 25 years
- 26 - 44 years
- 45 - 59 years
- 60 + years

Page Break

Q23 What is the state you live?

- New South Wales
- Victoria
- Queensland
- Western Australia
- South Australia
- Tasmania
- Northern Territory
- Australian Capital Territory

Page Break

Q24 What is your highest education level?

- Primary School
- Secondary School
- Vocational Education (TAFE and private training providers)
- University or Higher Education

Page Break

Q25 What is your total annual household income?

- $0 – $18,200
- $18,201 – $45,000
- $45,001 – $120,000
- $120,001 – $180,000
- $180,001 and over

**End of Block: Demographic Data**

**References**

Bovea MD, Ibáñez-Forés V, Pérez-Belis V, et al. (2018) A survey on consumers’ attitude towards storing and end of life strategies of small information and communication technology devices in Spain. *Waste Management* 71: 589-602.

Duke C, Thorun C, Dekeulenaer F, et al. (2018) *Behavioural study on consumers’ engagement in the circular economy – Final report.* {CEU}.

Genever M, Randell P and Baker B (2018) Victorian e-waste infrastructure network assessment report Reportno. Report Number|, Date. Place Published|: Institution|.

Habib H, Wagner M, Baldé CP, et al. (2022) What gets measured gets managed – does it? Uncovering the waste electrical and electronic equipment flows in the European Union. *Resources, Conservation and Recycling* 181: 106222.

Islam MT, Dias P and Huda N (2021) Young consumers’ e-waste awareness, consumption, disposal, and recycling behavior: A case study of university students in Sydney, Australia. *Journal of Cleaner Production* 282: 124490.

Pérez-Belis V, Braulio-Gonzalo M, Juan P, et al. (2017) Consumer attitude towards the repair and the second-hand purchase of small household electrical and electronic equipment. A Spanish case study. *Journal of Cleaner Production* 158: 261-275.

Shittu OS, Williams ID and Shaw PJ (2022) Prospecting reusable small electrical and electronic equipment (EEE) in distinct anthropogenic spaces. *Resources, Conservation and Recycling* 176: 105908.
